# Using social network analysis to examine inter-governmental relations in the implementation of the Ideal Clinic Realisation and Maintenance programme in two South African provinces

**DOI:** 10.1371/journal.pone.0251472

**Published:** 2021-05-12

**Authors:** Immaculate Sabelile Muthathi, Mary Kawonga, Laetitia Charmaine Rispel

**Affiliations:** 1 School of Public Health, Faculty of Health Sciences of the University of the Witwatersrand, Johannesburg, South Africa; 2 Department of Community Health, School of Public Health, Faculty of Health Sciences, University of the Witwatersrand, Johannesburg, South Africa; 3 Centre for Health Policy and South African Research Chairs Initiative, School of Public Health, Faculty of Health Sciences, University of the Witwatersrand, Johannesburg, South Africa; University of Cape Town, SOUTH AFRICA

## Abstract

**Background:**

Within the context of universal health coverage (UHC), South Africa has embarked on a series of health sector reforms. The implementation of the Ideal Clinic Realisation and Maintenance (ICRM) programme is a major UHC reform. Cooperative governance is enshrined in South Africa’s Constitution, with health a concurrent competency of national and provincial government. Hence, effective inter-governmental relations (IGR) are essential for the ICRM programme implementation.

**Aim:**

The aim of the study was to measure the cohesion of IGR, specifically consultation, support and information sharing, across national, provincial and local government health departments in the ICRM programme implementation.

**Materials and methods:**

Using Provan and Milward’s theory on network effectiveness, this study was a whole network design social network analysis (SNA). The study was conducted in two districts in Gauteng (GP) and Mpumalanga (MP) provinces of South Africa. Following informed consent, we used both an interview schedule and a network matrix to collect the social network data from health policy actors in national, provincial and local government. We used UCINET version 6.619 to analyse the SNA data for the overall network cohesion and cohesion within and between the government spheres.

**Results:**

The social network analysis revealed non-cohesive relationships between the different spheres of government. In both provinces, there was poor consultation in the ICRM programme implementation, illustrated by the low densities of seeking advice (GP = 15.6%; MP = 24.4%) and providing advice (GP = 14.1%; MP = 25.1%). The most cohesive relationships existed within the National Department of Health (density = 66.7%), suggesting that national policy actors sought advice from one another, rather than from the provincial health departments. A density of 2.1% in GP, and 12.5% in MP illustrated the latter.

**Conclusion:**

The non-cohesive relationships amongst policy actors across government spheres should be addressed in order to realise the benefits of cooperative governance in implementing the ICRM programme.

## Introduction

An explicit global target in the Sustainable Development Goals (SDGs) is the achievement of universal health coverage (UHC) by 2030 [1, 2]. UHC, which combines access to quality, essential health services and financial risk protection, is a major focus for policy-makers at a global level [3]. South Africa has also embarked on a series of health sector reforms towards UHC, with prioritisation of the implementation of the national health insurance (NHI) system [4]. The NHI is a health financing system that is designed to pool all health funds to provide access to quality, affordable health services for all South Africans irrespective of their socio-economic status [5]. The NHI is also designed to overcome the inequities between the public and private health sectors [5].

The first phase of implementing the NHI policy commenced in 2012, with a specific focus on strengthening of the public health sector [6]. The National Department of Health (NDoH) identified NHI pilot districts in all of South Africa’s nine provinces, and initiated several projects with the intention of overhauling service delivery in the public health sector [5]. These projects included but were not limited to the re-engineering of primary health care (PHC), improving public hospital infrastructure, and enhancing quality of care [7]. The NDoH prioritised the re-engineering of PHC, as the stated foundation of the South African health system [8]. Although the re-engineering of PHC consists of several strands, the notion of the “ideal clinic” took centre stage in the NDoH’s efforts to implement the first phase of the NHI [7]. The NDoH defined an ideal clinic as a clinic with “good infrastructure, adequate staff, adequate medicine and supplies, good administrative processes, with sufficient adequate bulk supplies and it uses applicable clinical policies, protocols and guidelines, and it harnesses partner and stakeholder support” [9]: page 11. The NDoH envisages the ideal clinics initiative, now called the Ideal Clinic Realisation and Maintenance (ICRM) Programme, as an approach to transform all PHC facilities in the country, in order to comply with the norms and standards prescribed by the Office of Health Standards Compliance (OHSC) [10]. The OHSC is a South African legal entity that aims to protect and promote the safety of health service users by ensuring compliance of all health establishments with the national core standards on quality [11]. The components of, and process towards reaching ideal clinic status have been described elsewhere [12].

Importantly, in 2015 the NDoH took primary responsibility for the implementation of the ICRM programme in the NHI pilot districts [10]. However, there are three conceptual and practical challenges with the implementation of the ICRM programme as a centrally driven health policy reform. Firstly, the South African Constitution lists health services as a concurrent functional area of both national and provincial levels [13]. The Constitution grants the national level the power to pass national legislation, set norms and standards, relate to international organisations and monitor the delivery of health care [13]. The Constitution also assigns responsibility to provinces for the planning and implementation of services. Although the Constitution grants local government the responsibility for the delivery of municipal health services, these services are defined as environmental health services in the National Health Act [13, 14]. However, in the large metropolitan municipalities, local government owns and manages PHC facilities, and employs health professionals. The legislative concurrency means that provincial health departments and those municipalities that provide PHC services should be involved in the conceptualisation, planning and the implementation of the various NHI projects or initiatives.

Secondly, the 2015 White Paper on the NHI is silent on the role of provincial and local government in the implementation of the NHI [7]. The 2017 NHI policy underscores the need to clarify the roles and responsibilities of the three spheres of government in health service delivery [5]. Thirdly, existing evidence suggests that effective inter-governmental relations (IGR) in the development of policies and strategies are essential for the success of major health sector reforms [15, 16]. IGR refer to “an interacting network of institutions at national, provincial and local government, created and refined to enable the various parts of government to work in a collaborative and coherent manner” [17]:1. Effective IGR facilitate decision-making, successful policy implementation, sharing of resources during policy implementation, optimal service delivery to citizens, and enhanced responsiveness to citizens [18–20].

However, in practice such federal arrangements across levels of government are complex and difficult to manage. A multi-country study on federalism and decentralisation in the governance, financing, administration or delivery of health care found significant variations in practices among the selected countries, influenced by country context, resource availability and human resource capacity [21]. The authors identified the need for further research on federalism, decentralisation, and health sector reforms [21]. There is also recognition that dysfunctional IGR have negative consequences for public health as has been found in the case of the suboptimal management of epidemics of infectious diseases [22], and in some instances could lead to the “joint-decision” trap [23]. A study on the implementation of the Affordable Care Act in the United States found that the law’s fragmented institutional design across federal and state governments created opportunities for persistent political contestation of the law, complicating its implementation [24]. Similarly, a study on long-term care reform in Italy found that the weak and uncertain legislative framework of federalism, combined with uncertainty on the allocation and distribution of resources and the delay in applying the equalisation mechanism contributed to policy implementation failure [25].

The interaction among stakeholders of the IGR network is also important. A study in China examined the inter-governmental and inter-organizational network of emergency response to major accidents [26]. Despite the criticality of collaboration among relevant stakeholders in emergency response, there were weak network relationships between private and public organisations at the central, provincial, and municipal government levels [26]. These weak IGR contributed to slow emergency response times.

In South Africa, the concept of cooperative governance is enshrined in the Constitution. Its stated purpose is to facilitate mutual decision-making, and ensure that the different government spheres support, communicate and consult with one another on relevant programmes [13]. In programme implementation, communication for sharing information, support and consultation is critical as it facilitates implementers’ buy-in, programme ownership and sustainability [27]. Studies on IGR in the implementation of health reforms are scanty, especially studies that use social network analysis. In 2014, Kawonga et al used social network analysis to examine the interactions between managers of disease control programmes and general health services [28]. The study found that there was insufficient communication and collaboration among human immunodeficiency virus (HIV) programme managers and district managers, who were their direct reports, and this had implications for monitoring and evaluation of the HIV programme implementation [28].

The NDoH reported that there was some stakeholder engagement in the implementation of the ICRM programme as the first phase of the NHI implementation [7, 10]. However, there is a dearth of empirical studies on IGR in the implementation of the ICRM programme. There are several reasons why IGR is important in the implementation of the ICRM programme. Firstly, health is a concurrent competency of national and provincial government, which implies that the Constitutional principles of cooperative governance [13] such as support, communication and consultation should be honoured in ICRM implementation. Secondly, the ICRM programme focuses on PHC, which is the foundation of the South African health system, and critical to the success of UHC reforms in the country [29]. Effective IGR could ensure successful ICRM programme implementation, thus contributing to improved health service delivery and responsiveness to citizens, as found in other studies [20, 30, 31]. Lastly, cohesive IGR enable sharing of limited resources, which is important in South Africa, given the challenges and constraints in the public health sector [32].

This study draws on Provan and Milward’s theory of inter-organisational network effectiveness and the tools of social network analysis to examine IGR in the ICRM programme implementation [33]. The specific aim of the study was to measure the cohesion of the relationships, specifically consultation, support and information sharing, amongst national, provincial and local government actors in the implementation of the ICRM programme.

## Methods

### Conceptual framework

Provan and Milward’s theory of inter-organisational network effectiveness posits that network effectiveness is influenced by both network structure and network context [33]. Network structure refers to centralised integration and direct, non-fragmented control, while network context refers to system stability and resource availability [33]. In this study, we examined the inter-organisational network structure in order to make inferences about the network effectiveness. We conceptualised the inter-organisational network as the interaction amongst health policy actors in national, provincial and local government who were involved in the ICRM programme implementation in the two South African provinces of Gauteng and Mpumalanga.

We examined network structure by measuring network density (a measure of cohesion), which is the extent to which members of a network interact with one another [34]. Density is a commonly used measure of collaboration in inter-organisational networks [35]. We also examined network structure by measuring network centralisation, which is the extent to which interactions amongst actors in a network are centralised i.e. revolve around one or a few individuals in the network [34].

### Study setting

The study was conducted in the NHI pilot districts of the two South African provinces of Gauteng (City of Tshwane) and Mpumalanga (Gert Sibande). The two provinces were selected purposively because of their geographical proximity to the researchers, logistical considerations and budgetary constraints.

The City of Tshwane district is a large metropolitan municipality in Gauteng Province (GP), where local government owns 24 of the 63 PHC facilities. Hence, in GP all three spheres of government are involved in ICRM programme implementation. The Gert Sibande district of Mpumalanga Province (MP) has 64 PHC facilities all managed by the provincial government [36]. Hence, in MP only national and provincial departments of health are involved in the ICRM programme implementation.

### Study design

This was a whole network study design that included all health policy actors eligible in the specified network boundary [37]. The network boundary consisted of all the health actors in national, provincial and local government involved in the implementation of the ICRM programme.

### Participant selection

The network boundary (inclusion criteria) for health policy actors were: involvement in conceptualisation of the ICRM programme; occupied a relevant health leadership position at national, provincial or local government sphere (e.g. chief director, director, deputy-director); and responsibility for the implementation of the ICRM programme in the NHI districts of Tshwane (GP) or Gert Sibande (MP). Implementation activities could include coordination, support, communication, training, ensuring availability of resources, and/or facility assessments. This ensured that the information for the social network analysis was obtained from the right individuals [38]. The principal researcher compiled a list of these actors, known as a network roster.

During data collection, snowball sampling was used to identify other potential ICRM network actors for inclusion on the network roster [39], provided that such person was mentioned by at least two actors. In the GP network, 22 actors were included, the national sphere was represented by four actors, and the City of Tshwane local government was represented by 6 actors. In the MP network, 26 actors were included, four actors represented national government, and 22 actors represented provincial government. In MP, local government does not provide PHC services, hence there were no actors involved in the ICRM programme.

### Development of the data collection instruments

Both an interview schedule and network matrix, i.e. a spreadsheet for plotting the existence or non-existence of relationships, were used to collect the data for social network analysis.

#### Interview schedule

The earlier sections focused on the context of the ICRM programme implementation, and the roles and responsibilities of government health departments in the ICRM programme, as this study was part of a larger doctoral research project [40]. The final section of the key informant interview schedule focused on IGR. This section contained six questions, which focused on consultation, support and information sharing.

In this study, consultation refers to either seeking or providing advice by the various policy actors in the ICRM programme implementation. Two questions asked participants to identify actors from whom they sought advice and those to whom they gave advice regarding implementation of the ICRM programme.

*Support* refers to a network actor providing encouragement, motivation and/or resources or technical assistance to another network actor in the ICRM programme implementation. Two questions asked participants to identify actors to whom they gave support and those from whom they obtained support regarding the implementation of the ICRM programme.

*Information sharing* refers to “sharing of tacit and/or explicit knowledge whether through formal documents or informal talks” [41]:4. In this study, there were two questions that asked participants to identify actors to whom they gave information and those from whom they received information regarding implementation of the ICRM programme.

#### Network matrix

The principal researcher (PR) developed a spreadsheet with columns and rows, to plot the presence and absence of a tie (relationship) for seeking advice, providing advice, seeking support, providing support, giving information and receiving information.

A team of health systems researchers reviewed the data collection tools for content validity. Following this review, the PR piloted the tools with two key informants from a different health district to ensure clarity of questions and the time taken to complete the interview. There were no revisions required.

### Data collection

Data collection for this study commenced in 2017. The PR contacted each key informant (i.e. identified actor) to request voluntary participation in the study. Following consent to participate, the PR arranged the interview date and time with each key informant. All interviews were conducted in English, after obtaining written, informed consent for both the interview and its audio-recording. The section on social network data was part of the interviews for the main study. The interview started with an introduction to the overall study, and an explanation of the voluntary nature of participation. Each participant was informed that there were two sections to the interview, with the last section focusing on interactions and relationships in the ICRM programme. On completion of the initial section, the PR introduced the key informant to the last section, which contained the IGR questions.

Following the introduction, the key informant was presented with a network roster, and was oriented to the list of actors on the roster. For example, the PR provided an explanation of the list of actors on the roster who were involved in the implementation of the ICRM programme. The key informant could use the list as a reminder of the various health policy actors and possible interactions during the implementation of the ICRM programme. The PR asked each question to the key informant, and captured all the data in the matrix. If a key informant reported to have spoken to a group or team instead of individuals, the key informant was encouraged to identify individuals within the group. The social network matrix was used for plotting the interactions as verbalised by the key informants (i.e. the health policy actors). The presence of a relationship was indicated by a ‘1’ and its absence was indicated by a zero ‘0’. The collection of the social network data took an average of 30 minutes, although the time varied depending on the individuals interviewed.

Before the end of each interview, the PR summarised the stated relationships to verify and validate that the relationships were captured correctly.

### Data management and analysis

All interview sessions were audio-recorded and transcribed verbatim. The PR read, and re-read summaries against the network matrix to validate data before analysis.

A total of 12 matrices of SNA data, six for each province, were cleaned in Microsoft Excel and imported to UCINET version 6.619 [42] for analysis. The data were analysed per province since the primary interactions were expected to be amongst actors within a province. We analysed the data (all 12 matrices for GP and MP) for the cohesion of the network structure i.e. the overall network cohesion and cohesion within and between the government spheres. Netdraw version 2.159 [43] software was used to generate sociograms.

Table 1 contains a description of terms and network measures that were analysed in this study.

**Table 1:** Network measures computed in the study.

Network Measures computed in the study	
Properties	Description
Number of possible ties	Possible ties are calculated as n (number of actors) multiplied by number of actors-1 (n (n-1))
Density	Density of a network is a measure of the number of present ties out of possible ties [44].
	It indicates the extent to which each actor is connected to all other actors in a network. Values ranges from 0 (no ties present) to 1 (all ties possible present) [35] expressed as a percentage in this study.
	Density can be categorised as low (below 30%), moderate (between 30 and 50%) or high (above 50%) [35].
Centralisation	The extent to which the network ties are focused on a few people in the network [34]. Values ranges from 0 to 1, closer to 0 means least centralised (i.e. there is almost equal distribution of ties, power, control etc.) and closer to 1 refers to highly centralised network (only a few central actors dominate interactions in the network [45].

#### Overall cohesion of the inter-governmental relationships

In order to determine the overall cohesiveness of the networks, the overall density of each network was computed by determining the number of present ties as a proportion of number of possible ties (number of ties if each actor were connected to all other actors in a network).

#### Network cohesion within each government sphere

We computed the densities for each government sphere in order to determine the cohesiveness of the network within that sphere. Density measured the extent to which actors within a government sphere interacted for consulting, supporting and sharing information with each other.

#### Network cohesion between government spheres

In order to determine the extent of interaction amongst actors across spheres of government, we computed cohesiveness of networks comprising actors between two spheres.

#### Ethical considerations

We obtained ethical approval from the Human Research Ethics Committee (Medical) of the University of the Witwatersrand (#M170661). Permission to conduct the study was obtained from the NDoH, the Gauteng and Mpumalanga provincial health departments, and the City of Tshwane local government health department. All participants were given a detailed information sheet, as well as a verbal explanation of the study. All participants were informed of the voluntary, confidential and anonymous nature of study participation. The study results are kept on a password-protected computer, and only the PR has access to the password.

## Results

**Fig 1:**
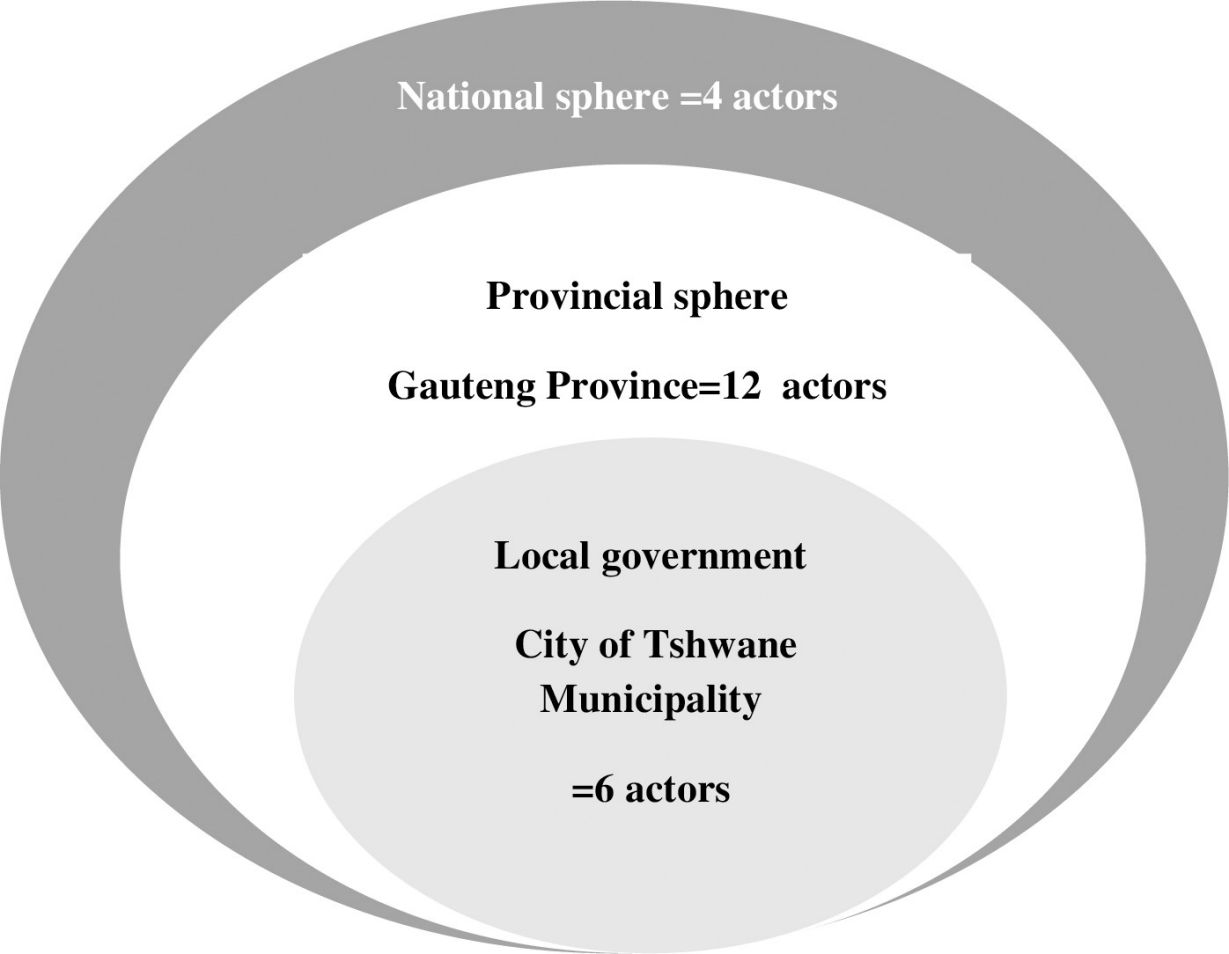
Overview of Gauteng province network.

**Fig 2:**
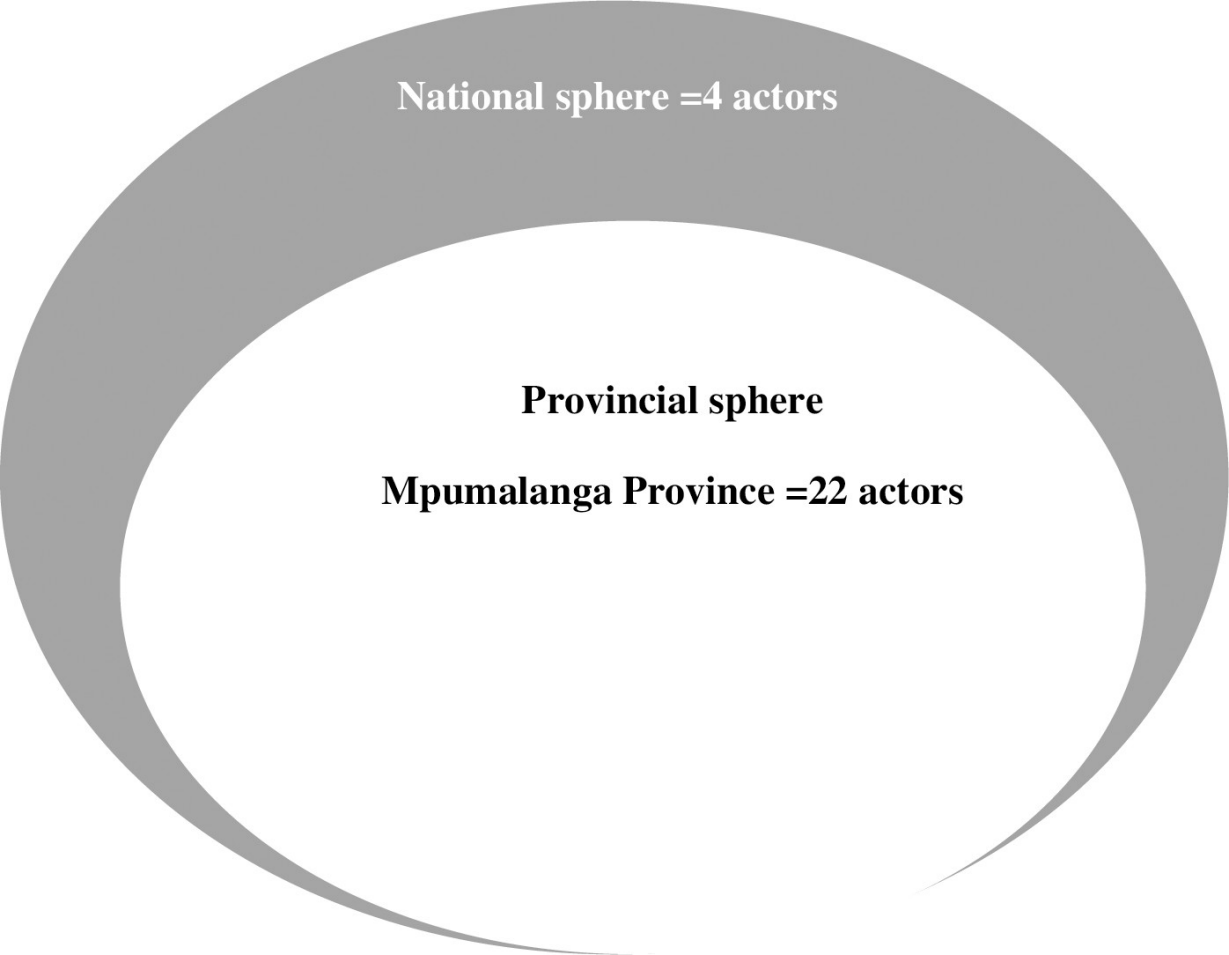
Overview of Mpumalanga province network.

We obtained a 100% response rate. Four actors represented the national government sphere, while MP was represented by 22 actors, GP by 12 actors, and the City of Tshwane local government was represented by 6 actors (Figs 1 and 2).

### Overall network cohesion

Table 2 shows the density scores for consultation (seeking or providing advice), support and information sharing for the network in GP. The network in Gauteng consisted of national, provincial and local government actors. In GP, the overall possible ties for GP were 462, and giving support obtained the highest density of 21.2%. In GP, the overall network for seeking advice had a density of 15.6%, and for receiving support a density of 15.2%.

**Table 2:** Overall network structure in Gauteng and Mpumalanga provinces.

Overall Network structure in Gauteng and Mpumalanga provinces												
Gauteng	n = 22 actors											
Mpumalanga	n = 26 actors											
	Seeking advice		Providing advice		Giving support		Receiving support		Receiving information		Providing information	
	GP	MP	GP	MP	GP	MP	GP	MP	GP	MP	GP	MP
#Possible network ties	462	650	462	650	462	650	462	650	462	650	462	650
#Existing ties in a network	72	152	65	163	98	156	70	151	47	130	50	163
Density (%)	15.6	23.4	14.1	25.1	21.2	24.0	15.2	22.3	10.2	20.0	10.8	25.1
Centralisation	0.6	0.7	0.6	0.6	0.5	0.5	0.5	0.6	0.6	0.6	0.8	0. 7

GP = Gauteng Province; MP = Mpumalanga Province

Table 2 also shows the network densities for consultation (seeking or providing advice), support and information sharing for the network in MP. The network in MP consisted of national and provincial government actors. In MP, the overall possible ties were 650, and the highest density of 25.1% was obtained for providing advice. The network for seeking advice had a density of 23.4%, and for receiving support a density of 22.3%. In both provinces, the interactions regarding the ICRM programme implementation revolved around a few individuals, illustrated by the centralisation values of 0.5–0.8 in GP and of 0.5–0.7 in MP.

**Fig 3:**
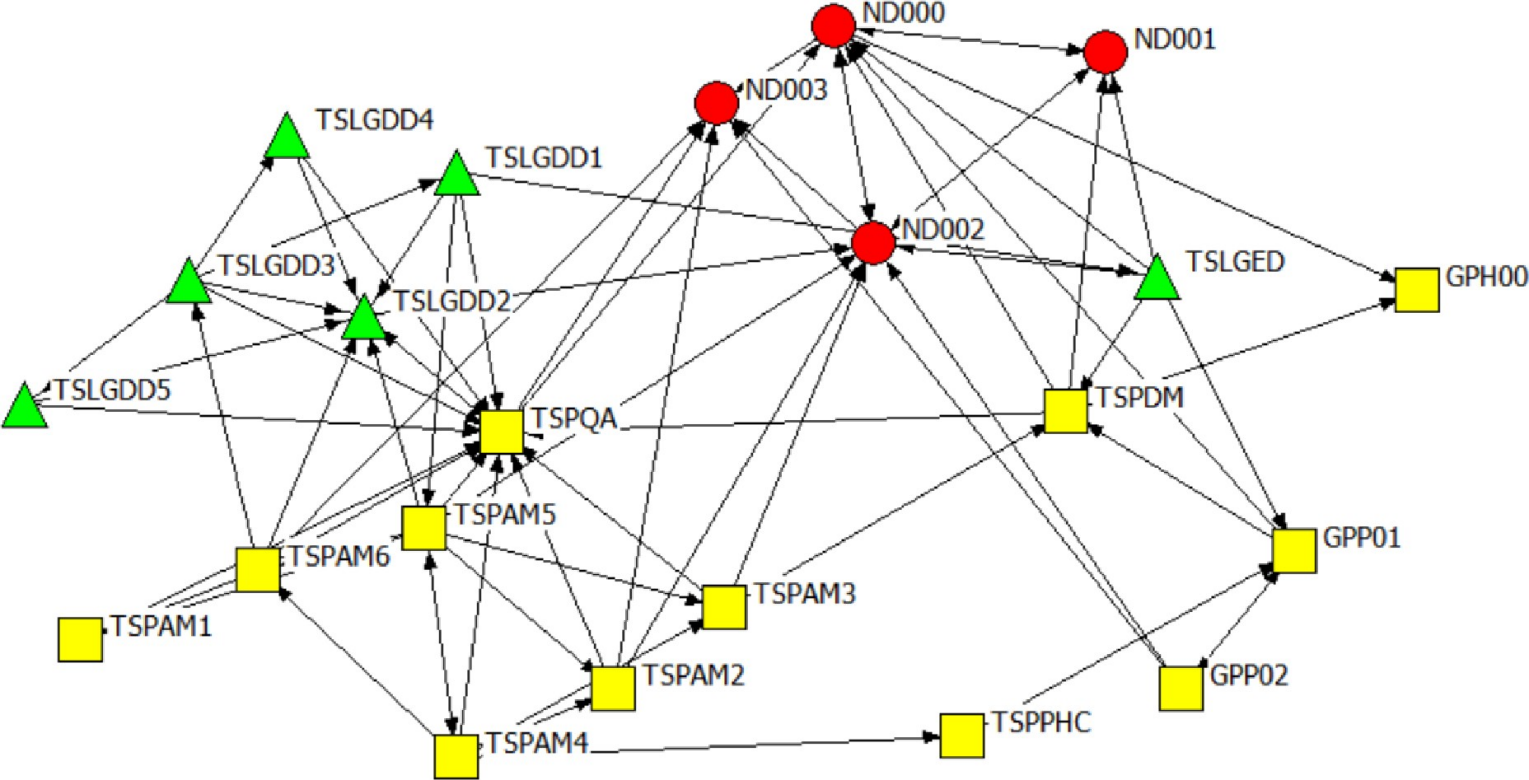
Gauteng province network: Seeking advice. Colour and Shape of nodes: red circle = National, yellow square = Provincial, and green triangle = Local Government. Nodes labels: ND = A label starting with TSP or GP refers to a provincial actor, TSLG = A label starting with TSLG refer to a local government actor.

**Fig 4:**
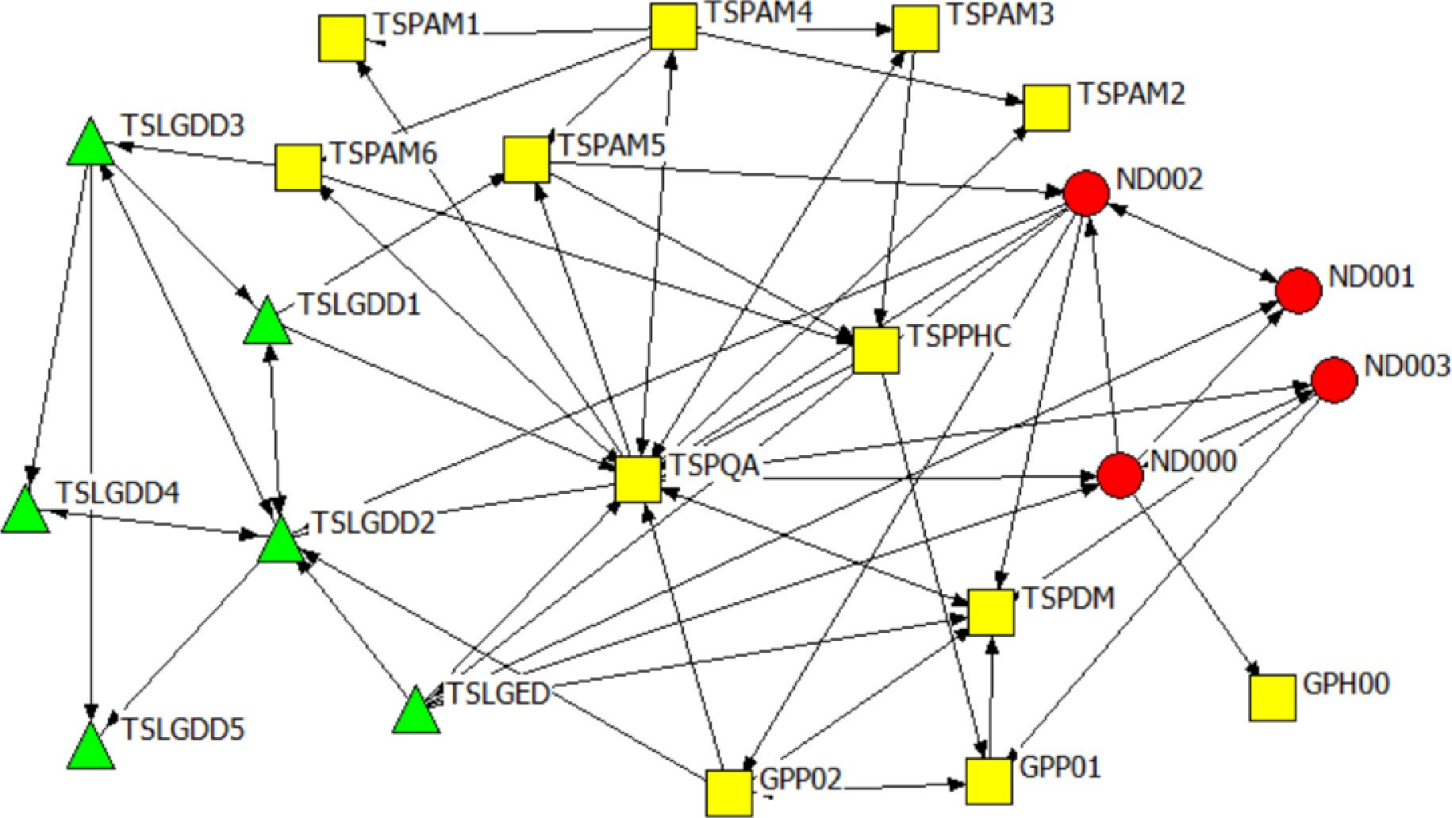
Gauteng province network: Providing support. Colour and Shape of nodes: red circle = National, yellow square = Provincial, and green triangle = Local Government. Nodes labels: ND = A label starting with TSP or GP refers to a provincial actor, TSLG = A label starting with TSLG refer to a local government actor.

**Fig 5:**
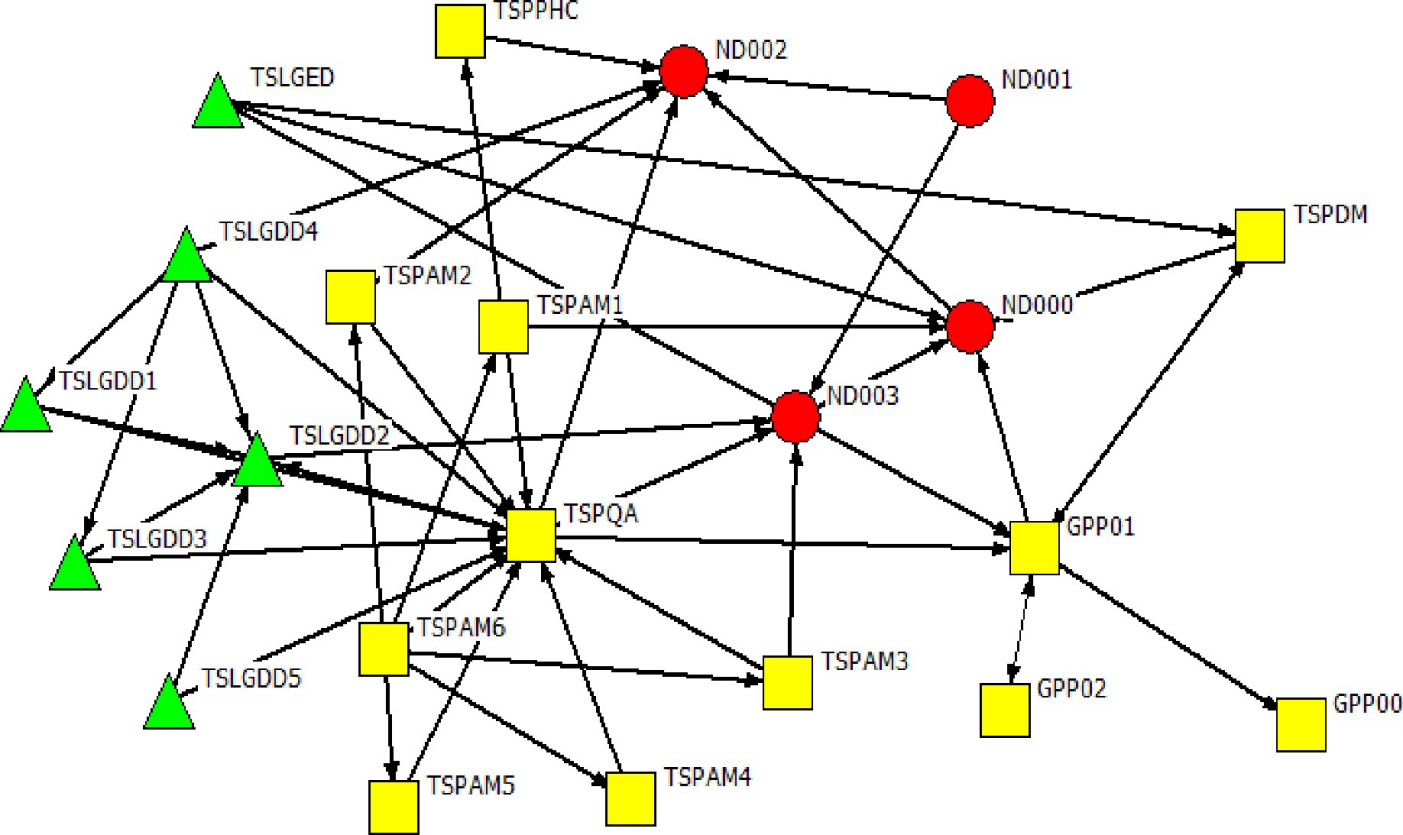
Gauteng province network: Receiving information. Colour and Shape of nodes: red circle = National, yellow square = Provincial, and green triangle = Local Government. Nodes labels: ND = A label starting with TSP or GP refers to a provincial actor, TSLG = A label starting with TSLG refer to a local government actor.

Figs 3–5 are the network diagrams visualising the interactions for seeking advice, providing support and for receiving information in GP. All networks appear sparse with some actors having no interactions with each other, within and across spheres (see unconnected nodes).

**Fig 6:**
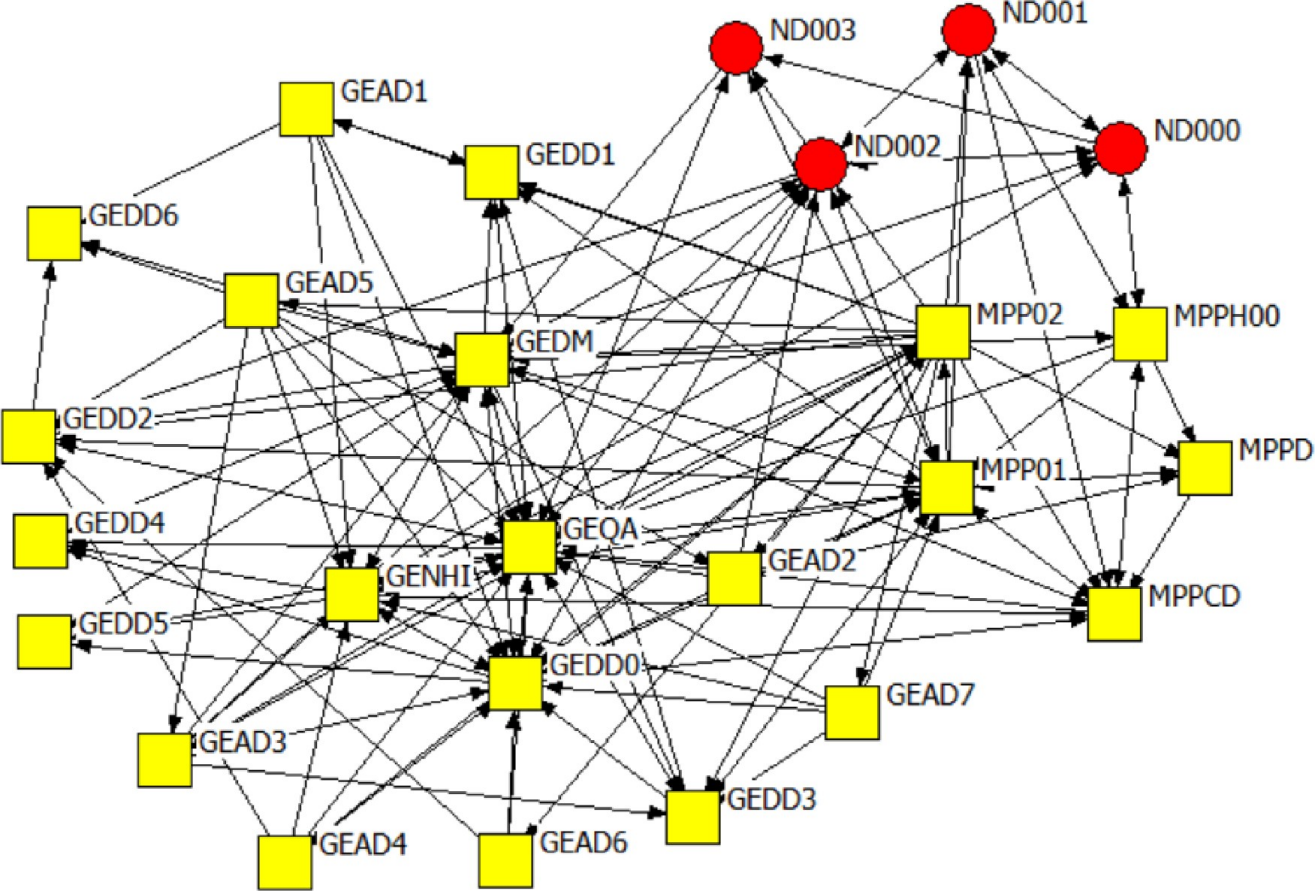
Mpumalanga province network: Seeking advice. Colour and Shape of the nodes: red circle = National, yellow square = Provincial. Nodes label: ND = A label starting with ND refers to national actors, MP & GE = A label starting with MP or GE refers to provincial actors.

**Fig 7:**
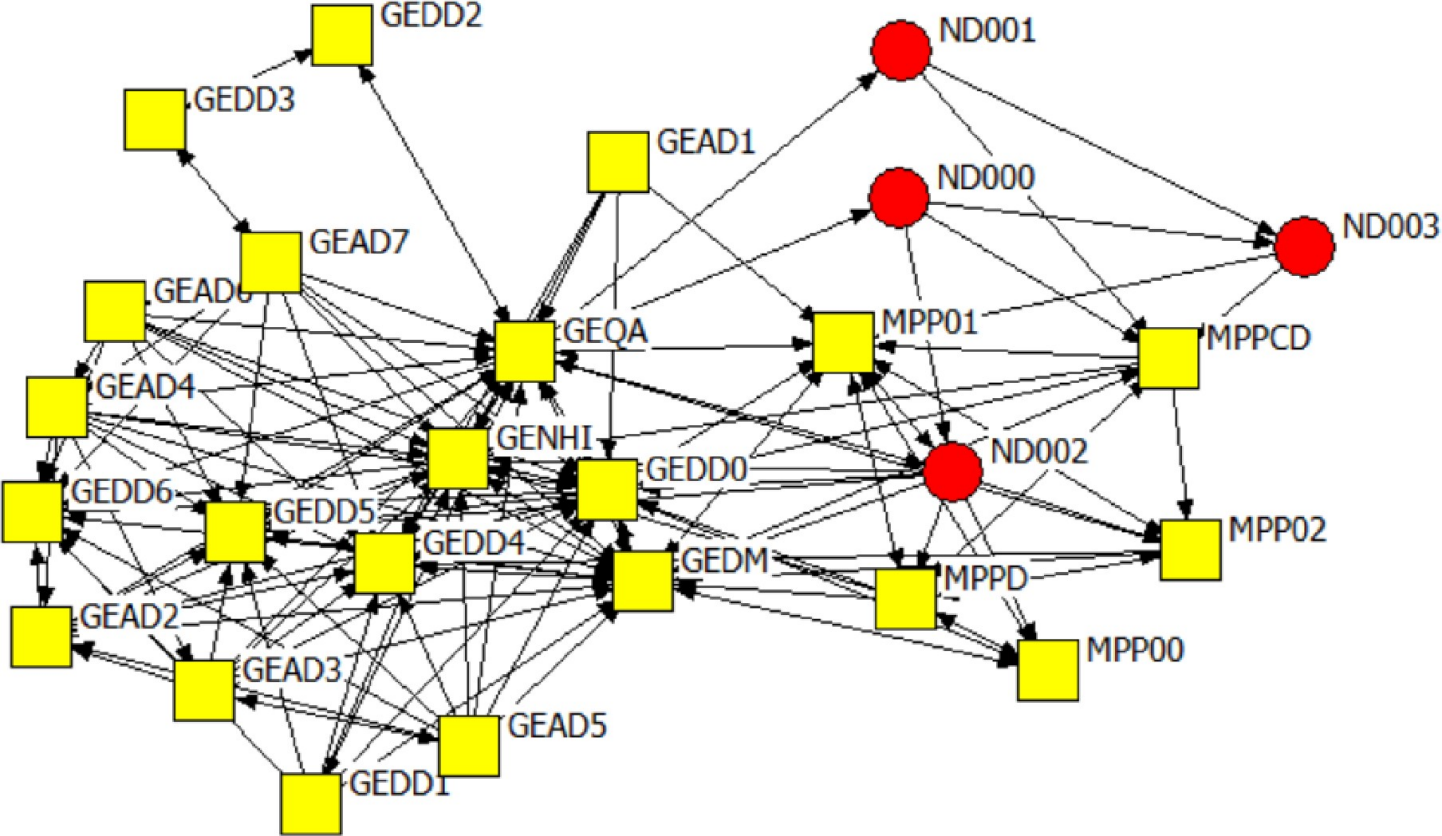
Mpumalanga province network: Providing support. Colour and Shape of the nodes: red circle = National, yellow square = Provincial. Nodes label: ND = A label starting with ND refers to national actors, MP & GE = A label starting with MP or GE refers to provincial actors.

**Fig 8:**
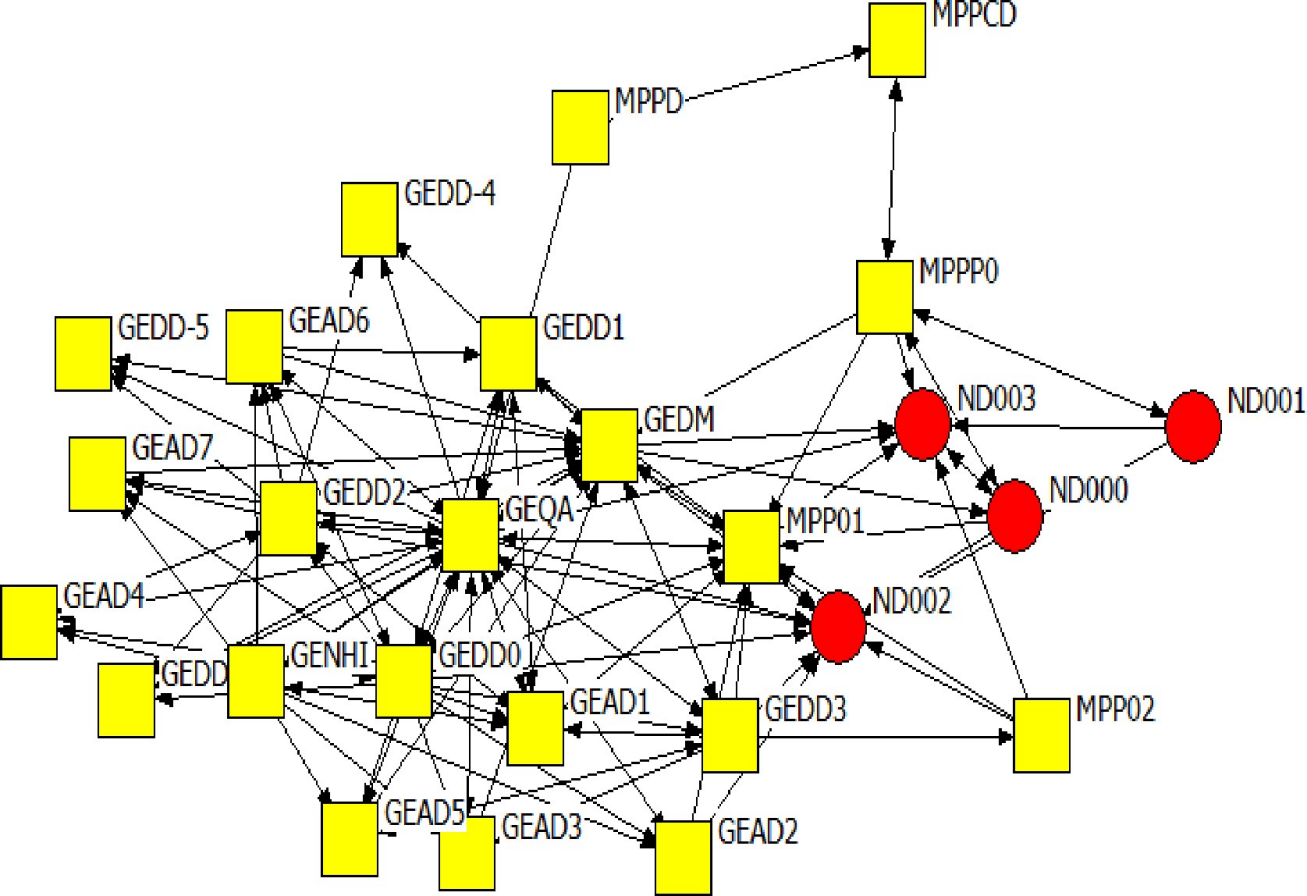
Mpumalanga province network: Receiving information. Colour and Shape of the nodes: red circle = National, yellow square = Provincial. Nodes label: ND = A label starting with ND refers to national actors, MP & GE = A label starting with MP or GE refers to provincial actors.

Figs 6–8 are the network diagrams visualising the interactions for seeking advice, providing support and for receiving information in MP. The MP networks appear less sparse than GP network, but also with actors having no interactions with each other, within and across spheres.

### Network cohesion within each government sphere

#### National sphere

Table 3 shows the density within the national sphere. The interaction with the highest density was for seeking advice (66.7%) amongst each other and the lowest density was for providing information to other actors within the national sphere (16.7%).

**Table 3:** Network cohesion within government spheres.

Network cohesion within government spheres				
	National government	Provincial government		Local Government
		GP	MP	
**Seeking advice**				
# Possible ties	12	132	462	30
# Observed ties	8	24	116	10
d (%)	66.7	18.2	25.1	33.3
**Providing advice**				
# Possible ties	12	132	462	30
# Observed ties	6	26	135	11
d (%)	50.0	19.7	29.2	36.7
**Giving support**				
# Possible ties	12	132	462	30
# Observed ties	5	53	134	13
d (%)	41.7	40.2	29.0	43.3
**Receiving support**				
# Possible ties	12	132	462	30
# Observed ties	3	25	131	14
d (%)	25.0	18.9	28.4	46.7
**Receiving information**				
# Possible ties	12	132	462	30
# Observed ties	3	16	104	6
d (%)	21.7	12.1	22.6	20.0
**Giving information**				
# Possible ties	12	132	462	30
# Observed ties	2	20	141	5
d (%)	16.7	15.2	29.0	16.7

GP = Gauteng Province; MP = Mpumalanga Province; d = density

#### Provincial sphere

Table 3 shows the density scores for the consultation (advice), support, and sharing information networks within each of the selected provinces. The densities (d) for the seeking advice networks within the provincial sphere were 18.2% in GP and 25.1% in MP. GP had moderate density for providing support within the provincial sphere (d = 40.2%). Both provinces had low densities for receiving information from policy actors within the provincial sphere, d = 12.1% in GP and 22.6% in MP.

#### Local government

Within the local government sphere in the City of Tshwane in Gauteng, the actors had moderate interaction for seeking advice (d = 33.3%) and for receiving support from each other (d = 46.7%), but had low densities (d = 20.0%) for sharing information amongst themselves (Table 3).

### Cohesion between government spheres

Table 4 shows the density scores for networks of actors across two spheres. For seeking advice, the relationships between national and provincial sphere had low densities of 2.1% in Gauteng and 12.5% in Mpumalanga. For giving support, the density was 18.8% in GP and 16.1% in MP. For receiving information, the densities were 2.1% for GP and 4.5% for MP.

**Table 4:** Network cohesion between government spheres.

Network cohesion between spheres of government–density (%)												
	Seeking advice		Providing advice		Giving support		Receiving support		Receiving information		Providing information	
	GP	MP	GP	MP	GP	MP	GP	MP	GP	MP	GP	MP
National to Provincial	2.1	12.5	14.6	19.3	18.8	16.1	2.1	14.9	2.1	4.5	14.6	17.2
National to Local Government	0.0		28.3		16.7		0.0		0.0		4.2	
Provincial to National	27.1	18.2	6.3	5.7	4.2	41.7	25.0	4.5	16.7	18.2	8.3	5.0
Provincial to Local Government	5.6		5.6		9.7		5.6		1.4		9.7	
Local Government to National	16.7		8.3		8.3		16.7		12.5		0.0	
Local Government to Provincial	11.1		5.6		4.2		9.7		9.7		5.6	

GP = Gauteng Province; MP = Mpumalanga Province

#### National and local government spheres

There were no ties for national sphere seeking advice from local government. The network for giving support had a density of 16.7%, and for giving information had a density of 4.2%.

#### Provincial and local government spheres

The network density on seeking advice was 5.6%, on giving support was 9.7%, and providing information was 4.2%.

## Discussion

Drawing on Provan and Milward’s theory of the multi-dimensional nature of networks [33], a key objective of this study was to examine the cohesion of inter-governmental relations (IGR), specifically consultation (seeking or giving advice), support and information sharing in the implementation of the Ideal Clinic Realisation and Maintenance (ICRM) programme. Cohesion of IGR is important when implementing a national policy across multiple levels of government (in this case the ICRM programme) because it enhances the sharing of values, knowledge, and resources [33, 44, 46], and contributes to the achievement of common goals [33]. Network cohesion indicates collaboration among policy actors, resulting in their commitment to implementation, a degree of uniformity in implementation, and long-term sustainability [16, 46, 47].

In both Gauteng and Mpumalanga provinces, the social network analysis (SNA) revealed that there was poor consultation amongst actors in the ICRM programme implementation, shown by the low densities of seeking advice and providing advice. Within the national department of health (NDoH), the density for seeking advice was 66.7%, and 50.0% for providing advice to each other. This is in contrast to consultation network densities of 2.1% for the NDoH seeking advice from Gauteng province (GP) and 12.5% for the NDoH seeking advice from Mpumalanga province (MP). This means that national policy actors primarily consulted with one another, rather than with the provincial actors, who are the main implementers of the ICRM programme. The slight difference in densities could be due to the geographical location, as Mpumalanga is further located, and is more rural province, and may have required additional efforts in interaction. Although the density scores for the NDoH providing advice to Gauteng and Mpumalanga provinces were slightly higher at 14.6% and 19.3% respectively, the apparent low level of consultation is concerning. This is because inputs from provincial and local government actors, who are the implementers, are critical for their buy-in and success of the programme. Within Gauteng province, this pattern of insufficient consultation across spheres of government was repeated, with a low density for the province seeking advice from local government and for providing advice to local government. This is despite the fact that local government continues to provide PHC services in Gauteng and the City of Tshwane municipality (local government) had 24 PHC facilities involved in the implementation of the ICRM programme. The City of Tshwane reported on the progress of ICRM programme in their integrated development plan [48]. Hence, consultation with local government was critical in the implementation of the ICRM programme.

Similarly, the densities for providing and receiving support within all networks ranged from low to moderate, with the highest densities in the local government sphere. The SNA shows that the densities of support networks spanning across spheres of government were lower. The support interactions between national and provincial government spheres had maximum densities of 18.8% in Gauteng and 16.1% in Mpumalanga, while the density for support interactions between the Gauteng provincial health department and the City of Tshwane local government health department was only 9.7%. Inadequate support had a negative impact at primary health care facility level, most acutely experienced by the facility managers who incurred penalties for non-compliance with prescribed standards that were the responsibility of the national and/or the provincial departments of health [12].

Information sharing in the implementation of the ICRM programme was poor, illustrated by the low densities for receiving information and for providing information in all IGR networks, and within government spheres. Our SNA study found low densities in the two provincial and local government health departments for receiving information from the NDoH. This could be an indication that information flow across spheres of government remains hierarchical.

The overall picture suggest non-cohesive or fragmented IGR, illustrated by the low densities on consultation, support and information sharing These weak IGR could partly explain the difficulties experienced in the sustainability of the ICRM programme, with a reversal of ideal clinic status by primary health care facilities in both provinces [49]. In the 2018/19 financial year, 89% of clinics in Gauteng and 46% of clinics in Mpumalanga achieved ideal clinic status [49]. At face value, Gauteng is the best performing province in achieving ideal clinic status [49]. However, this is a decrease from 93% of clinics with ideal clinic status, achieved in 2015/16 [50]. The NDoH expressed concern about provincial variations in achieving ideal clinic status, and about the reversal of progress with the many clinics that have lost ideal status since 2015/16 [49]. However, in the proposed NDoH recommendations for dealing with these problems, there is little attention paid to the policy implementation processes of consultation, support, and information sharing. This would need to be addressed to ensure long-term sustainability of the ICRM programme.

Several South African studies have highlighted the negative influence of insufficient consultation and poor communication on policy implementation [51–53]. Although the contexts and methodologies differ from the SNA used in this study, studies in other countries of different levels of development have also highlighted the negative consequences of non-cohesive networks. A study in the United States (US) reported that conflicts between federal and state governments created difficulties in adopting and implementing proposed reforms of the Medicaid program, which provide health insurance coverage for people with low incomes [24]. This resulted in discrepancies in service coverage across the various states [24]. Another US study highlighted the contestations between the federal and state governments in the implementation of Affordable Care Act because of lack of consultation, despite the federal government provision of financial support and incentives for implementation [54]. In Italy, a study found that the challenges of inter-governmental relationships combined with policy fragmentation and fiscal constraints contributed to the failed implementation of long-term care policies [25].

Studies in other African countries have also highlighted the problems of weak IGR. A Ghana study that examined the implementation of an eye care programme in 12 districts across different government levels found that actors at sub-district level experienced difficulties in distributing resources, and they felt excluded from receiving information on progress [55]. In Nigeria, a qualitative study on the management of IGR highlighted weak communication and poor relationships, characterised by competition, conflict and confrontation, which in turn influenced goal achievement [20]. In Kenya the implementation of the free maternity service was rushed with inadequate consultation, which reported inconsistencies in implementation [56].

In this study, the SNA measured high centralisation values of 0.5–0.8 in Gauteng and 0.5–0.7 in Mpumalanga. This means that the consultation, support and information sharing on ICRM programme implementation revolved around a few individuals in each province, rather than a more distributed leadership of the programme. Provan and Milward’s theory suggests that the network of interaction should be centralised [33] in order to enhance coordination for goal achievement [33]. However, centralised networks have disadvantages of undue reliance on certain individuals, potential manipulation of policy design, the unequal distribution or withholding of network resources, and information, and inconsistencies in implementation [34]. The high degree of centralisation, combined with a top down approach from the NDoH posed potential risks for the ICRM programme implementation. These risks include lack of buy-in from front-line implementers, lack of commitment, and lack of sustainability.

Various studies found that these risks played out in different country contexts, with negative consequences for policy implementation [57–59]. In Brazil, a study on decision-making in an inter-governmental health forum found that the Ministry of Health dominated decisions, with little consideration of the implementation issues raised by the municipal and state government, and subsequent policy implementation failures [57]. In Nigeria, a 2012 study that used social network analysis (SNA) to examine the implementation of the vaccination programme, found a disjuncture between the federal, centralised network structure and the implementers who were at the periphery, that exclusion of implementers in decision-making process discouraged their sense of ownership of the programme. [58]. Similarly, a SNA study on Hepatitis C policymaking in Iran found that domination of decision-making by national level policymakers coupled with poor stakeholder involvement hindered implementing actors’ cooperation in the implementation process [59].

The study findings have implications for IGR in South Africa’s ongoing health reforms. This is because health services are a concurrent responsibility of both national and provincial departments of health, necessitating collaboration [13]. Firstly, the NDoH should take the lead in improving IGR in the implementation of the ICRM programme. This could be done through optimal utilisation of existing forums, such as the National Health Council and/or its technical committees. Existing forums could be used for consultation, information sharing, and building capacity of relevant policy actors. Secondly, the NDoH should guide the development of a clear implementation plan from the bottom-up. This will ensure that all relevant stakeholders are involved, that their concerns are taken into account, as well as the contextual factors that influence ICRM programme implementation. The implementation plan should include strategies for capacity building, communication, mechanisms for informing and engaging with stakeholders, and indicators for monitoring and evaluation.

This study was one of the first studies that used SNA to measure the cohesion of the IGR network in the implementation of a primary health care reform. However, the cross sectional nature of our study provides a snapshot of the experiences of study participants during 2017, when the data were collected. The measurement of densities on consultation (advice), support and information sharing might be different when measured in 2020. Nonetheless, the 2019 report on the ICRM programme [49] suggests that the findings of the study on the weak IGR, and low densities remain relevant, and should be taken into account in improving ICRM programme implementation. Hitherto, ICRM programme implementation has focused mostly on addressing staff, infrastructure and financial constraints [49]. However, Provan and Milward’ theory underscores the multiple interactions that comprise full networks (in this case national, provincial and local government), and the implementation of public policy through networks of [health] service providers, who need to cooperate with one another [60]. Hence, building IGR in consultation, support and information sharing, must be a critical element of implementation.

The study participants were self-reporting which could have introduced social desirability bias of either over-or- under-reporting. This was mitigated by leaving the SNA questions to the final section of the interview, after the principal researcher established rapport with the individuals. In addition, there was no intrinsic incentive to misrepresent the relationships, as all individuals are in senior government positions. The potential limitation of error due to poor recall was also minimised by using the network roster which listed all possible actors [37]. The study was conducted in two provinces and two national health insurance districts, and cannot be generalised to other provinces and/or districts. Future studies should aim to get a national picture on IGR, and compare IGR across districts and provinces.

The study combined SNA with Provan and Milward’s network theory to examine the overall network cohesion and cohesion within and between the spheres of government. The study findings could provide the baseline for similar studies that measure densities and centralisation of IGR in the implementation of health sector reforms, whether in South Africa, or other low and middle income country settings.

## Conclusion

The study has generated new knowledge on inter-governmental relations (IGR) in the implementation of the Ideal Clinic Realisation and Maintenance (ICRM) programme in two South African provinces. The non-cohesive relationships amongst policy actors in the national, provincial and local government spheres could impede the implementation of the ICRM programme. Cohesive IGR provide benefits, such as leveraging resources, mutual learning, capacity building, shared risks, opportunity for innovation, increased responsiveness and increasing accountability to communities [61–63]. The South African Constitution enables cooperative governance and strong IGR. Health policy implementation requires the various actors to consult with one another, share information and resources, and provide mutual support. Although the findings are specific to South Africa, this study adds to the knowledge on IGR and health sector reforms, and provides lessons for other low-and middle-income countries embarking on health sector reforms that require cohesive IGR.
